# Early medieval vernacular Celtic glosses: originals or translations?  A case study on the Vienna Bede

**DOI:** 10.12688/openreseurope.16006.1

**Published:** 2023-07-05

**Authors:** Bernhard Bauer

**Affiliations:** 1 Institute Centre for Information Modelling - Austrian Centre for Digital Humanities, Karl-Franzens-Universitat Graz, Graz, Styria, 8010, Austria

**Keywords:** Glossing Traditions, Old Irish, Early Medieval Celtic Languages, Early Medieval Latin, Computus, Venerable Bede, Multilingualism

## Abstract

This study investigates the Old Irish glossing tradition on the Venerable Bede’s De Temporum Ratione, a computistical work from the early eighth century. Its main source is the Vienna Bede, a fragmentary manuscript with Old Irish and Latin glosses dating from the late eighth/early ninth centuries. It focuses on parallel glosses found in the Gloss-ViBe corpus where the Vienna Bede has an Old Irish gloss and the other manuscripts feature glosses in another language (Latin or Old Breton/Welsh). Minute analysis of individual glosses is used to determine whether early medieval vernacular Celtic glosses originals or translations from Latin glosses? The heterogenic nature of early medieval gloss corpora makes this a complex question for which there is no straightforward answer: for some glosses, a translation from Latin into Irish is almost inevitable, but others suggest Irish influence on the Latin parallel glosses. Accordingly, each case is discussed individually and the results are synthesised in the final part of the article.

## Plain language summary

In 725 the English monk Bede wrote the Reckoning of Time, a book about calculating time and making a calendar. ^1^ This was mainly important to solve a complicated mathematical problem: what is the date of Easter? The work was widely distributed. Early medieval scholars annotated their copies. These annotations are also called glosses and are a great source of information about contact between people in this period. Glosses appear in different languages, e.g., Latin, or the Insular Celtic languages, i.e., Old Irish, Old Breton or Old Welsh. As the manuscripts were copied the glosses were also copied and sometimes translated into a different language. This helps us to understand the history of the annotations on Bede's Reckoning of Time. This study looks at one manuscript, the Vienna Bede, which dates to the late eighth or early ninth centuries. It compares it to three other manuscripts from around the same time. The comparisons show how closely linked the four manuscripts are. Some of the parallels can even show which language the glosses were translated from. Translations can go from Latin to the Celtic languages and the other way around.

## Abbreviations

**Table:** 

Ang.	Angers, Bibliothèque municipale 477
BCr.	Karlsruhe, Badische Landesbibliothek, Augiensis pergamentum 167 (olim Codex Augiensis CLXVII)
BVi.	Vienna, Österreichische Nationalbibliothek, Codex 152985 (olim Suppl. 2698)
CCSL 123B	*Corpus Christianorum Series Latina*, i.e. Jones, 1977
DTR	*De Temporum Ratione* by the Venerable Bede
Gloss-ViBe	*Early Medieval Glosses And The Question Of Their * *Genesis:* *A Case Study On The Vienna Bede*, i.e. Bauer, 2021–2023
Gr.	Ancient Greek
Lat.	Latin
MS	manuscript
OBret.	Old Breton, c. 800-1100
OIr.	Old Irish, c. 700-900
OW	Old Welsh, c. 800-1100
Sg.	St Gall, Stiftsbibliothek, MS 251

## Introduction

Discussing the Old Irish glosses on Priscian’s Latin grammatical textbook, Moran (2015, 136) made this thought-provoking statement “We may [...] question whether the Irish glosses are original compositions at all, or merely translations from inherited Latin sources.” Previous studies on these matters, e.g., Lambert (1983), Killion (1992) or Bauer (2019d), have shown that there is no straightforward answer to this complex question and minute analyses of individual glosses need to be carried out. They have demonstrated that there are examples for translations from Latin to Old Irish but also the other way around. For the glosses found in St Gall, Stiftsbibliothek, MS 904 – a copy of Priscian’s *Ars Grammaticae* – I have argued elsewhere that “not all of the Old Irish glosses […] are mere copies of original Latin glosses” ( Bauer, 2019d, 17).

Is this also true for the Old Irish glosses on the Venerable Bede’s *De Temporum Ratione* (= DTR)? The English monk's computistical *opus magnum* is commonly dated to 725 (cf. Wallis, 2004, xvi fn. 4). It is about measuring time and constructing a calendar. And computus is, after all, “nothing more than a complicated mathematical problem: how to find the date of Easter” ( Wallis, 2004, xviii). Bede’s work was widely distributed in the early medieval period; since these are complicated matters, it was accordingly heavily glossed. Within the bulk of transmitted manuscripts there are also important sources for early medieval Celtic languages (see the sources below) and in what follows, I will concentrate on those. To find answers to the question raised in the title, I am focussing on glosses in parallel transmission in the present study. Parallel glosses are annotations on the same lemma of the base text transmitted in different manuscripts. The first list of Celtic parallel glosses on DTR – concentrating mainly on the vernacular glosses of Angers 477 (Old Breton/Welsh) and the Karlsruhe Bede (Old Irish) – was offered by Lambert (1983, 121–127). He identifies the following levels of parallel transmission ( Lambert, 1983, 120):

- linguistic borrowings from Irish to Breton
- glosses with the same contents over the same lemmas in the base text; in this case the parallelism sometimes goes as far as word-by-word translation/transposition
- glosses with the same contents which appear in different locations of the base text.

Lambert concluded that a good portion of Angers’ commentary (especially the glosses in hand B) originates from an Irish tradition. ^2^ The present study goes one step further back in the Celtic glossing tradition on Bede’s *De Temporum Ratione*. It takes the Vienna Bede (= BVi.) as its main source, to research the genesis of the Old Irish glossing tradition on Bede’s computistical work which influenced the Angers 477’s (vernacular) glosses to a large extent. Accordingly, I am focussing on parallel glosses with a vernacular Old Irish gloss in BVi. (the oldest of the four manuscripts of the corpus – see below) and glosses in another language (Latin or Old Breton/Welsh) in (at least one of) the other three manuscripts.

## Methods

The study consists of two main parts: the chapter *corpus and analysis* presents and scrutinises the data. The parallel glosses as well as the lemma they are glossing in the base text are analysed using the historical-comparative method from philology and historical-linguistics. The two final chapters are synthesising the results and hence discuss the genesis of the vernacular glosses in the Vienna Bede – the main research question of this paper.

## Sources

The source manuscripts for this article are identical to those of the Gloss-ViBe corpus ( Bauer, 2021–2023), which is available under https://gams.uni-graz.at/query:glossvibe.allglosses. This corpus consists of all the glosses in the Vienna Bede and their parallels found in three other manuscripts. The manuscripts were chosen, because they share a high number of parallel glosses.

1. **Vienna,** **Österreichische Nationalbibliothek, Codex 152985 (olim Suppl. 2698)** dates from the late 8 ^th^/early 9 ^th^ century. The fragmentary manuscript – only four folios have survived – transmits (parts of) twelve chapters of Bede’s *De Temporum Ratione* ^3^. There are glosses in Latin and Old Irish. Images of the manuscript are available online from the Austrian National Library ^4^ and in the facsimile view of Gloss-ViBe ^5^.
2. **Angers, Bibliothèque municipale 477** either originates from Brittany or North-East France ^6^. A calculation found on folio 21a dates it to 897 (cf. Lambert, 2005). This composite manuscript contains, inter alia, Bede’s *De Temporibus, De Temporum Ratione,* and *De Natura Rerum*. There are glosses in Latin, Old Breton/Welsh, and “bretonised” Old Irish (cf. Lambert, 2005). A digital facsimile of the manuscript is online at the Bibliothèque Virtuelle des Manuscrits Médiévaux. ^7^
3. **Karlsruhe, Badische Landesbibliothek, Augiensis pergamentum 167 (olim Codex Augiensis CLXVII)** dates to the first half of the ninth century (cf. Bronner, 2013). Among other computistical works, it features *De Temporibus*, *De Temporum Ratione*, and *De Natura Rerum*. Glosses on the base text appear in Latin and Old Irish. Digital images of the manuscript are available via the Badische Landesbibliothek. ^8^
4. **St Gall, Stiftsbibliothek, MS 251** also dates to the first half of the ninth century. ^9^ It contains Bede’s *De Natura Rerum* and *De Temporum Ratione* in full, and the ending of *De Temporibus* – all annotated in Latin. These glosses are closely related to the ones found in BCr. (cf. Bauer, 2019c). High-resolution scans are available via the *e-codices* project. ^10^

## Data collection

For the present paper I have narrowed down data from the Gloss-ViBe corpus to all those instances in which there is an Old Irish gloss in the Vienna Bede that has a parallel gloss in at least one different language – Latin of Old Breton/Welsh – in one of the other three manuscripts mentioned in the sources ( Bauer, 2023).

## Corpus and analysis

The presentation of the corpus is based on the layout I have established for Bauer (2019b). The parallel glosses are consecutively ordered as they appear in the Vienna Bede. They are arranged according to the DTR chapters they are found in. The base text is quoted from Corpus Christianorum Series Latina edition (= CCSL 123B) by Jones (1977) and its translation by Wallis (2004). The glosses and their translations are presented as they are recorded in Gloss-ViBe. Abbreviations, however, are silently expanded in this paper. The only other editorial intervention is that Old Irish verbal forms are presented in the standardised form which is used in Stifter *et al.* (2021).

### VII. De Nocte

CCSL 123B, 297

*Et quomodo nocte* ^DTR 1^ *caeca procul accensas faces intuens cirumposita quaeque loca eodem lumine perfundi non dubitas, tametsi* ^DTR 2^ *tenebris noctis obstantibus non amplius quam solas facium flammas cernere praeualeas […] sidera quidem ipsa lucem radiantia parent* ^DTR 3^ *[…] Lunam uero aiunt cum infimas sui circuli apsidas ^DTR 4^ plena petierit, nonnumquam umbra memorata fuscari*
If on a dark night, you are positioned at a distance from some blazing torches, you see some of the surrounding area suffused with their light, although the darkness of night is all about, and all you can see are the separate flames of the torches themselves […] the stars themselves appear to be shining lights […] ( Wallis, 2004, 29)

DTR 1:

**Table:** 

**BVi. 1a9b1**	*dorchai*
	‘dark’
**Ang. 50a2a**	*or timuil*
	‘of the darkness’

For an analysis of the Old Irish gloss of BVi. I refer to my discussion in Bauer (2017, 31–32). While the Old Irish gloss features an adjective, the Old Breton parallel gloss in Ang. has a noun phrase consisting of preposition plus article and a noun meaning ‘darkness’.

DTR 2:

**Table:** 

**BVi. 1a11.3**	*ce nid·aciam-ni*
	‘although we do not see (it)’
**Ang. 50a3**	*cenit-guelhum-ni* ^11^
	‘although we do not see it’

Lambert (1983, 127) lists these glosses as an example of Old Breton glosses resembling their Old Irish parallels word for word. The conjunction, negative particle, infixed pronoun, verb proper, and emphatic particle are transposed one-to-one. As we shall see below, there are also such examples in Latin and Old Irish.

DTR 3:

**Table:** 

**BVi. 1a16.4**	*adrigiter*
	‘by which they appear’
**BCr. 27a56**	*apparent*
	‘they appear’

This is an interesting example, because the verbal form Lat. *apparent* ‘they appear’ in BCr. 27a53 is very similar to the glossed lemma of the base text: Lat. *parent* ‘they appear’. In fact it only has an added preverb Lat. *ad* ‘to, towards’ which – on the surface – does not significantly change the semantics. The Old Irish gloss BVi. 1a16.4 is also interesting, because it features a third person plural indicative relative verb OIr. *ardrigiter* ‘by which they appear’ although there is no relative construction found in the base text. Both glosses are therefore a bit puzzling. The Latin one does not seem necessary and the Old Irish one is somewhat odd because of its grammatical analysis. What is furthermore noteworthy is that the Karlsruhe Bede also features a form of OIr. *ardraigidir* later in the manuscript where the third person present indicative OIr. *ardrigid* glosses once again Lat. *parent*, but in this case, it is not relative.

DTR 4:

**Table:** 

**BVi. 1a18.5 **	*fithissi absida .graece. circulus interpretatur*
	‘circular courses; in Greek, circle is translated as apside’
**Ang. 50a7c**	*amestidiou*
	‘circular courses’
**BCr. 27b3**	*.id est. absida graece interpretatur lucida …* ^12^
	‘i.e. in Greek absida is translated as bright […]’
**Sg. 55.32**	*id est. absida graece interpretatur lucida*
	‘i.e. in Greek absida is translated as bright’

When comparing these parallel glosses it makes sense to divide the bilingual gloss in BVi. into two parts. The first one consists of only one word OIr. *fithissi* meaning ‘circular courses’ or ‘orbits’, which has a parallel in the Old Breton gloss *amestidiou* ‘id.’ found in Ang. While the gloss does not continue in the latter manuscript the second part of BVi. 1a18.5 – although not verbatim – is also found in the other two manuscripts. The latter two are quotes from Isidore’s *Etymologiae* (Book 15, 8, 7): *Absida Graeco sermone, Latine interpretatur lucida, eo quod lumine accepto per arcum resplendeat.* ^13^ BVi. 1a18.5 imitates the Isidorian quote, but states that Greek *absida* means circle. While Gr. αψίδα is usually only used to denote the architectural structure, the related form Gr. ἁψῑ́ς can also mean ‘wheel, hoop, disc’. A connection with circle is therefore not too far-fetched. In addition to the explanation in DTR 4, there is also the marginal gloss BVi. 1a19a which states *[…] absida […] graecus et interpretatur splendida* ‘absida […] Greek and it is interpretated as bright’. This one also picks up on Isidore’s *Etymologiae*, but features Lat. *splendida* ‘bright’ instead of *lucida*.

CCSL 123B, 298

*… discursandi ubique ac uictum quaeritandi* ^DTR 5^ *copia suppeteret …*
in order that they may have an opportunity to go about and seek their food ( Wallis, 2004, 298)

DTR 5:

**Table:** 

**BVi. 1a41.6**	*con·destis*
	‘that they should seek’
**BCr. 27b28**	*.i. quaerere*
	‘i.e. to seek’

As I argue in a forthcoming work ^14^, these parallel glosses are one of the few instances in which a direction of borrowing can be securely determined. The argument that the Old Irish gloss in the Vienna Bede is a translation from an original Latin gloss which is attested in Karlsruhe is based on the fact that the grammatical construction in the former – third person plural past subjunctive in a nasalising relative construction – is usually not used to gloss Latin gerunds like the glossed lemma *quaeritandi* ‘to be sought’. Such constructions are, however, used when translating the Latin infinitive, a concept alien to Old Irish grammar. And indeed, BCr. 27b28 features the Latin infinitive *quaerere* ‘to seek’.

### VIII. De Hebdomada

CCSL 123B, 300

*Sex diebus operaberis et facies omnia opera tua; septimo autem die* ^DTR 6^ *sabbati domini Dei tui non facies omne opus.*
Six days shalt thou labour and do all thou hast to do; but on the seventh day, the sabbath of the Lord thy God, thou shalt do no work. ( Wallis, 2004, 32)

DTR 6:

**Table:** 

**BVi. 1b27.10**	*fo chosmailius septimi diei mundi*
	‘in the likeness of the seventh day of the world’
**Ang. 50b12a**	*similitudine septimi diei initii mundi*
	‘in the likeness of the seventh day of the world's beginning’
**BCr. 27c22**	*ad similitudinem .uii. diei*
	‘in the likeness of the seventh day’

The beginning of the gloss features the Old Irish phrase *fo chosmailius* ‘in the likeness’ in the Vienna Bede. In BCr. a similar construction is transmitted but in Latin *ad similitudinem* ‘in (the) likeness’. The gloss in Ang. differs in two ways, because it only has the ablative singular of the Latin word for ‘likeness’ (omitting the preceding preposition) and also introduces Lat. *initii* ‘of the beginning’ in the second part of the gloss. The glosses in BVi. and BCr. seem to be translations from each other. Unfortunately, it is not possible to determine a direction for the translation. 

CCSL 123B, 302

*Ferias* ^DTR 7^ *uero habere clerum primus papa Siluester edocuit*
Pope Silvester was the first to instruct the clergy to have *feriae* [weekdays] ( Wallis, 2004, 34)

DTR 7:

**Table:** 

**BVi. 1c24b.14**	*.i. lanre sechtmaine*
	‘the full space of a week’
**Ang. 51a6e**	*dies ebdomadae*
	‘days of the week’

The gloss in Ang. states the *feriae* are the days of the week, whereas the Old Irish gloss in the Vienna Bede stresses the fact that Pope Silvester instructed the clergy to have weekdays for ‘the full space of a week’ i.e. every single day. Therefore, a connection is possible, but not necessary.

CCSL 123B, 303–304

*Tertia species hebdomadis in celebratione penetcostes agitur, vii uidelicet septimanis dierum et monade* ^DTR 8^ *, hoc est quinquaginta diebus, impleta […] sancta sanctorum intrabat annuis antea fructibus, hoc est frumenti, uini, et olei, ex ordine *collectis** ^DTR 9^ […] (304) *prima, tertia et septima die iubebantur lustrari* ^DTR 10^
A third kind of week occurs in the celebration of Pentecost; it is completed in 7 times 7 weeks, plus one, which is 50 days. […] enter the Holy of Holies after the year’s fruits of grain, wine and oil had first been collected in order […] to be purified on the first, third and seventh day ( Wallis, 2004, 35)

DTR 8:

**Table:** 

**BVi. 1c38.16**	*uno .i. ond oenfiur*
	‘one i.e. from one man’
**Ang. 51a16e**	*.i. una die dominico*
	‘one Sunday’
**BCr. 27d44**	*uno die*
	‘one day’
**Sg. 57.37b**	*i. uno die*
	‘i.e. one day’

The gloss in BVi. falls into two parts, a Latin one and an Old Irish one which are separated by the Tironian note for ‘id est’. While the second part does not have a parallel in the other three manuscripts the first one – Lat. *uno* ‘one’ – occurs in all three of them. It explains the meaning of *monade* found in the base text. Lat. *monas* goes back to Gr. μονάς ‘unit’. The gloss in BVi. simply notes that ‘one’ is meant with *monade*. BCr. and Sg. add the word for day and the longest gloss, i.e. Ang. 54a16e, states that the 50th day (mentioned in the base text) is a Sunday. This suggests that the length of (the parts of) the Latin glosses and therefore also their complexity proceed from simple in the Vienna Bede to (more) complex in the other manuscripts. Similar evolutions have also been noted for other glosses in the Celtic glossing tradition on Bede (cf. Bauer, 2019c).

DTR 9:

**Table:** 

**BVi. 1d5b.17**	*do idbart*
	‘for offering’
**Ang. 51a22b**	*.i. sacrificio*
	‘(for) sacrifice’

With a single phrase these two glosses explain that the goods were collected ‘for offering’. Since Old Irish does not have an ablative case, the BVi. gloss resorts to a prepositional construction with the preposition OIr. *do* ‘to, for’ plus the dative singular of OIr. *idbart* ‘(act of) offering, sacrifice’, the verbal noun of OIr. *ad·opair* ‘offers, sacrifices’. Ang. 51a22b features the ablative singular of Lat. *sacrificium* ‘something made sacred or given to a deity, sacrifice’. Since the glossed lemma of the base text Lat. *collectis* is also in the ablative case, this does not help to detect the direction of translation.

DTR 10:

**Table:** 

**BVi. 1d10.18**	*no·glandis*
	‘that they should be cleansed’
**Ang. 51a26a**	*mundari*
	‘cleansed’
**BCr. 28a4**	*.i. mundari .uel consecrari. uel lauari.*
	‘i.e. cleansed or consecrated or washed’
**Sg. 58.2**	*.i. mundari*
	‘i.e. cleansed’

As already mentioned for DTR 5 above, infinitives are a grammatical category alien to Old Irish. Therefore, glossators had to find other ways of explaining them to Irish-speakers. In BVi. 1d10.18, the Latin present passive infinitive *lūstrārī* (from Lat. *lūstrāre* ‘to purify, circle, wander over, illuminate’) is translated by the third person plural past subjunctive passive of the verb OIr. *glanaid* ‘to cleanse, purify, purge’. The other three manuscripts all have Lat. *mundari,* also a present passive infinitive (of *mundāre* ‘to clean, cleanse’). BCr. offers another two passive infinitives with synonyms meaning ‘to be consecrated, dedicated, cleansed, washed’.

### VIIII. De Hebdomadibus Septuaginta Propheticis

CCSL 123B, 305

*… embolismos uero menses, qui de annuis xi epactarum diebus adcrescere solent, non lege patria tertio uel altero anno singulos adiciens* ^DTR 11^ *…*
[…] did not include in the second or third years (as tradition decrees) the embolismic months which normally accumulate from the eleven days of the epact of every year. ( Wallis, 2004, 36)

DTR 11:

**Table:** 

**BVi. 1d26b.18b**	*.i. indeud ogdato ocus circuil*
	‘i.e. in the end of the octad and the [entire] cycle’
**Ang. 51a26b**	*.i. ogdad*
	‘i.e. octad’
**BCr. 28a25**	*ogdad et endicad*
	‘octad and endicad’

This is an unusual case, because compared to the other two manuscripts, BVi. has the longest gloss and hence also offers the most information (cf. the discussion of DTR 8).

### XI. De Mensibus

CCSL 132B, 315

*Quia uidelicet luna, quae praesenti* ^DTR12^ *anno, uerbi gratia, per nonas Maias septima decima existit*
For the Moon, which this year, for instance, is in its 17 ^th^ day on the nones of May [7 May] ( Wallis, 2004, 43)

DTR 12:

**Table:** 

**BVi. 2a28.19a**	*.i. noe*
	‘i.e. Noah’
**BCr. 29b4**	*in quo fuit noe*
	‘in which Noah was’

Although DTR 12 is not an example of a vernacular/Latin parallel gloss, it is worth discussing here. Because so far BVi. 2a28.19a has been read as Old Irish *i·mbe* ‘in which you may be’, a nasalising relative construction ^15^ featuring the preposition *i* ‘in’ and the second singular present subjunctive of the substantive verb. This would have somehow fitted the Latin gloss found in BCr., which has the preposition *in*, the relative pronoun *quo* and also a form of the substantive verb. The latter however is the third singular perfect indicative ‘he/she/it was’, which is in concordance with the nominative singular *Noe* ‘Noah’. The last word of BCr. 29b4 led me to the new (and most likely correct) reading of the parallel gloss. There is a crease in the manuscript at the beginning of BVi. 2a28.19a which makes the beginning of the gloss a bit uncertain, but it is very likely the Tironian note *.i.* ‘i.e.’. The rest of the gloss *noe* is, however, clearly readable. Hence, this example shows the importance of researching glossing traditions as a whole, because only with the help of parallel glosses can certain glosses be understood and correctly interpreted. The base text of chapter XI talks about the age of the moon on the nones of May and gives Noah’s ark as an example, because that’s the date that Noah went into the Ark. Bede goes on to say that the moon is in its 17 ^th^ day on the nones of May [May 7] and it will be 27 days old on the day before the nones of May in the following year. The two glosses tell the reader that Bede is talking about the year in which Noah went into the Ark. This stands in opposition to the interpretation of modern scholarship. It is the common opinion nowadays that Bede is actually talking about his present. ^16^ Indeed, the passage is one of the examples that is used to date the composition of DTR, because the moon would have been 17 days old on May 7 in the year 722.

CCSL 123B, 316

*Notandum sane quod nimium falluntur qui mensem definiendum uel ab antiquis definitum autumant quamdiu luna zodicaum circulum peragit, que nimirum, sicut diligentior inquisition naturarum edocuit, zodiacum quidem xxvii diebus et xiii horis, sui uero cursus ordinem xxviiii diebus* ^DTR 13^ *et xii horis, salua sui saltus ratione, conficit.*
Note well that those who say that the month ought to be defined, or was defined by the ancients, as the length of time in which the Moon traverses the zodiacal circle, make a serious mistake. As more painstaking inspection of nature has taught, the Moon plainly completes the zodiac in 27 days and 8 hours, but its proper course is 29 days and 12 hours, setting aside the calculation of the “leap of the Moon”. ( Wallis, 2004, 43–44)

DTR 13:

**Table:** 

**BVi. 2a35.22**	*.i. reim ṅgrein*
	‘the course of the sun’
**Ang. 53b11a**	*ad solem sequendum tamen*
	‘however, to follow the sun’

Although the two glosses bear very similar semantics and appear at the same position within Bede’s textbook, the exact connection is not clear.

CCSL 123B, 318

*Quem decimo kl. Septembrium die terminantes, residuos quinque dies epagomenas* ^DTR 14^ *uel intercalares siue additos uocant…*
this [last month] ends on the 10 ^th^ kalends of September [23 August], and they call the remaining five days *epagomena – “intercalated” or “added”* ( Wallis, 2004, 45)

DTR 14:

**Table:** 

**BVi. 2b28.25**	*forescaidi*
	‘superlunar’
**BCr. 29c1**	*super lunares quia mene luna interpretatur et epo super*
	‘superlunar because “mene” means “luna” and “epo” “super”’
**Sg. 63.21**	*i. super lunares quia mene luna interpretatur et epo super*
	‘i.e. superlunar because “mene” means “luna” and “epo” “super”’

The gloss in BVi. only consists of the nominative plural *forescaidi* ‘superlunar’. This *hapax legomenon* is a compound of the preposition OIr. *for* ‘on, upon, over’ and the adjective OIr. *éscaide* ‘lunar’ – an adjectival formation to OIr. *éscae* ‘moon, month’. The same concept is rendered in Latin in BCr. and Sg. with the preposition Lat. *super* ‘on, upon, over’ plus the adjective *lūnāris* ‘lunar’. The substitution of Lat. *super* with OIr. *for* is commonly found in the glosses and also in the present corpus. In DTR 21, BVi. 4b10.50 and BCr. 32a57 have the Old Irish preposition with a Roman numeral *for xi* ‘on the eleventh’ where Ang. 58a11b has *super xi* ‘id.’ (see below) ^17^. While OIr. *forescaidi* is the only word of the gloss in BVi., the parallel gloss in the other two manuscripts offers an Isidorian etymology for the glossed lemma *epogomena*. This goes back to Gr. ἐπαγόμενος ‘added on’ and is used for the five extra days which are added to a year in the Egyptian calendar to make each year last 365 days. Only the etymology presented in BCr. 29c1 and Sg. 63.21 helps to understand the Old Irish gloss in the Vienna Bede. It states that *epogomena* means ‘superlunar’ because *mene* (= Gr. μήνη) means Lat. *luna* ‘moon’ and *epo* (= Gr. ἐπῐ́) means Lat. *super* ‘on, upon, over’. Since annotating *epogomena* with ‘superlunar’ only makes sense when one knows of this made-up connection, it seems plausible that the Old Irish gloss in BVi. is a translation of the Latin found in the other two manuscripts rather than the other way around.

### XII. De Mensibus Romanorum

CCSL 132B, 325

*Verum una re a Graecis differebant, nam illi confecto ultimo mense, Romani non confecto Februario sed post uicesimum et tertium diem eius, intercalabant, terminalibus* ^DTR 15^ *scilicet iam peractis.*
On one point, in fact, they differed from the Greeks, for while they intercalated after the end of the last month, the Romans intercalated, not at the end of February, but after its 23 ^rd^ day, that is, when the Terminalia was over. ( Wallis, 2004, 50)

DTR 15:

**Table:** 

**BVi. 3a29.32**	*feli .i. termini* ^18^
	‘feasts i.e. of Terminus’
**Ang. 54abis22a**	*festis termini*
	‘feasts of Terminus’
**BCr. 30a50**	*id est feris termini hic est plutonis*
	‘i.e. feasts of Terminus; this is of Pluto’
**Sg. 67a8a**	*.i. feriae terminalis.*
	‘i.e. of the terminal feast’

Although not using the exact same words, all glosses feature the phrase ‘feasts of Terminus’. In contrast to BVi., Ang. and Sg., BCr. has a more elaborate gloss which also features *hic est plutonis*. It is also noteworthy that in Ang. a part of a marginal gloss a few lines above (Ang. 54abis19f) features a bilingual Old Breton and Latin version of the Old Irish gloss found in BVi.: *ante in dies interkalationis fiebant guilou termini* ‘before in the days of intercalation the feasts of Terminus took place’. Lambert (1983, 122) only records the final two words of this gloss and accordingly connects the two Celtic glosses although they do not appear on the same lemma of the base text.

### XIII. De Kalendis, Nonis, Et Idibus

CCSL 123B, 325–326

*Priscis temporibus pontifici minori haec prouidentia delegabatur ut nouae lunae primum obseruaret aspectum uisumque* (326) *regi sacrificulo* ^DTR 16^ *nutiaret.*
In olden times, the responsibility for observing the first appearance of the new Moon and of announcing its sighting to the royal sacrificing-priest was delegated to a minor priest. ( Wallis, 2004, 50–51)

DTR 16:

**Table:** 

**BVi. 3a36.33**	*don primsacard*
	‘to the chief priest’
**Ang. 54abis27a**	*.i. maiori pontifici*
	‘i.e. to the greater high-priest’
**BCr. 30b1**	*.i. regi colenti sacrificia hoc est regi qui sacrificiis perfiebat uel pontifici maiori.,*
	‘i.e. to the king who performs the sacrifices, that is, to the king who was appointed to the sacrifice or to the greater high-priest’
**Sg. 67.13b**	*i. regicolenti sacrificia hocem* (sic!) *qui sacrifitas prefiebat uel pontificimaiori*
	‘ i.e. to the king who performs the sacrifices, that is, to the king who was appointed to the sacrifice or to the greater high-priest’

As I have in stated elsewhere ( Bauer, 2019c, 41) the glosses in BVi. and Ang. are very likely connected and might even be translations of each other. A direction for this translation, however, cannot be established.

### XVIIII. Item De Eodem Si Quis Computare Non Didicit

CCSL 123B, 343–344

*Et ut diebus quo signare uolebamus literae sufficerent, non singulis has diebus* (344) *sed alternis apposuimus* ^DTR 17^
In order that there might be enough letters for the days we wish to indicate, we have not placed them against every day, but against every other day. ( Wallis, 2004, 63)

DTR 17:

**Table:** 

**BVi. 4a10.42**	*.i. da lae fri oien litir*
	‘i.e. two days with one letter’
**Ang. 57b16e**	*dou did cum unam literam*
	‘two days with one letter’
**BCr. 31d54**	*.i. da llae for óen littir*
	‘i.e. two days on one letter’

I have already dealt with these three glosses in Bauer (2017, 37–38) and I refer you to the detailed discussion there.

CCSL 123B, 344

*… aperto codice* ^DTR 18^ *nota literam quae eidem sit praeposita diei* ^DTR 19^ *…*
[…] open the [calendar-]codex, and note the letter prefixed to that day. ( Wallis, 2004, 63)

DTR 18:

**Table:** 

**BVi. 4a2a.43**	*felere*
	‘calendar’
**Ang. 57b26a**	*guilerou*
	‘calendars’
**BCr. 32a12**	*.i. félire*
	‘i.e. calendar’

Stifter (2022) has recently discussed the interconnections of OIr. *félire* and OBret. *guiler, guilerou, guileri* and OW *gueleri* all bearing the same semantics ‘calendar’. He concluded that the latter are “learned, erudite adaptations […] of OIr. *félire*” ( Stifter, 2022, 4). DTR 18 is, therefore, not necessarily an example of direct translation/transposition from Old Irish to Old Breton, but reflects common practice.

DTR 19:

**Table:** 

**BVi. 4a23b.44**	*.i. i·mbí*
	‘in which you are’
**Ang. 57b26d**	*in quo es*
	‘in which you are’
**BCr. 32a13b**	*.i. i·mbi*
	‘in which you are’

The glosses annotate Lat. *diei* ‘day’ in what reads in Wallis' (2004, 63) translation: “[s]o if you wish to know what sign or what part of the month the Moon is in on any given day of the year, open the [calendar-] codex, and note the letter prefixed to that day.” Both Stokes and Strachan (1901–1903) and Stifter *et al.* (2021) translate/analyse the form of the substantive verb OIr. *·mbi* in BVi. and BCr. as third person singular habitual present. In the light of the parallel gloss in Ang. this breakdown should be reconsidered. In analogy to Lat. *es* ‘you are’ in Ang. 57b26d, the verbal form in the Old Irish glosses should also be interpreted as second person singular habitual present. All three glosses therefore directly address the reader. While a connection between the Latin and the Old Irish glosses is very likely, the direction of translation is, unfortunately, uncertain. 

CCSL 123B, 344–345

*Cirumfer oculos ad latera – hinc Geminorum* *ex*(345) *trema* , *illinc* ^DTR 20^ *Iunii mensis initia deprehendes esse notata.*
Now run your eye along to the margins. You will discover that the end of Gemini is noted on one side and the beginning of the month of June on the other. ( Wallis, 2004, 64)

DTR 20:

**Table:** 

**BVi. 4a34.46**	*dind leth ailiu*
	‘from the other side’
**Ang. 57b34a**	*an parth alall*
	‘on the other side’
**BCr. 32a28**	*.i. dind leith ailiu*
	‘i.e. from the other side’

What is interesting in this instance are the different prepositions used in the two languages: ‘on’ vs. ‘from’. The nouns also differ, but this might be caused by the fact that the native cognate of OIr. *leth*, OBret. *let* is only attested in compounds (cf. Fleuriot, 1964, 241).

### XX. Quota Sit Luna In Kalendas Quasque

CCSL 123B, 346

*Si uis scire quota est luna in kl. iunias anno tertio, tene regulares xii, adde epactas* ^DTR 21^ *anni illius xxii, fiunt xxxiiii. Tolle xxx, remanent iiii. Quarta est luna in kl. memoratas. Quod si quis obiecerit* ^DTR 22^ *uel huius uel praecedentis argumenti alicubi ordinem ordinem uacillare…*
If you want to know what Moon it is on the kalends of June in the third year, take the regular 12, add the epact for that year – 22 – and that makes 34. Subtract 30, and 4 remain; on the kalends in question, the Moon is four days old. Should anyone object that the order of either this or the preceding formula is shaky at any point […] ( Wallis, 2004, 66)

DTR 21:

**Table:** 

**BVi. 4b10.50**	*for xi*
	‘on the eleventh’
**Ang. 58a11b**	*super xi*
	‘on the eleventh’
**BCr. 32a57**	*.i. for xi.*
	‘i.e. on the eleventh.’

As already mentioned in the discussion of DTR 14 above, the Celtic-speaking practice of putting prepositions in front of dates can be also seen in the Latin gloss found in the Angers manuscript.

DTR 22:

**Table:** 

**BVi. 4b12.51**	*hi frithcheist*
	‘in objection’
**Ang. 58a13b**	*s. contra dixerit*
	‘i.e. it contradicted’
**BCr. 32b1**	*.i. hi frithcheist*
	‘in objection’

The glosses in the two languages entertain different strategies. The Latin one in Ang. has a verb in the third person singular – just like the glossed lemma. The Old Irish glosses on the other hand have a noun phrase consisting of preposition plus noun.

CCSL 123B, 347

*Sunt autem anni tres circuli decemnouenalis in quibus idem argumentum stabilitatem* ^DTR 23^ *sui tenoris conseruare nequeat…*
However, there are three years in the 19-year cycle when this formula cannot preserve the stability of its course […] ( Wallis, 2004, 67)

DTR 23:

**Table:** 

**BVi. 4b24.56**	*.i. ar ni·toscelai argumint acht bliadni slain*
	‘i.e. for the argument only ascertains a whole year’
**Ang. 58a22a**	*quia non explorat argumentum nisi annum saluum*
	‘because it only explores the argument of the whole year’
**BCr. 32b19**	*.i. ar ni·tosceli argumint acht bliadni slaín..*
	‘i.e. for the argument only ascertains a whole year’

In this case it is interesting to note that the Old Irish and Latin glosses exactly mirror each other: conjunction, negative particle, verb, noun, conjunction, noun, adjective. Something that – as already mentioned above – Lambert (1983, 127) has detected for Old Breton and Old Irish parallel glosses.

CCSL 123B, 347

*Item anno* ^DTR 24^ *xviiii, quia luna embolismi tertio die nonarum Martiarum incipit*
Again in the nineteenth year, because the Moon of the embolismic [month] begins on the 3 ^rd^ nones of March [5 March] ( Wallis, 2004, 67)

DTR 24:

**Table:** 

**BVi. 4b36.57**	*forcenn*
	‘end’
**Ang. 58a32**	*finis circuli est*
	‘it is the end of the cycle’
**BCr. 32b32**	*.i. forcenn noidecdi*
	‘i.e. end of the nineteen-year cycle’

This example illustrates very well the problems of fragmentation and the poor condition of the Vienna Bede: a part of folio 4 is cut off after *forcenn*. Therefore, we do not know whether the gloss continued on like in BCr. or originally only had the Old Irish word for ‘end’. Comparing the glosses found in Ang. and BCr., we see that in contrast to the Latin one in Angers which states that it is the end of the cycle, the Old Irish one in the Karlsruhe Bede stresses that Bede talks about the nineteen-year cycle, something that is also mentioned in the base text.

CCSL 123B, 348

*Si enim ipsum argumentum iuxta Aegyptios a Septembrio mense ubi principium est anni eorum inchoaueris, necesse est ut luna Iulii mensis eo anno xxviiii dies ut numquam alias habeat, uno uidelicet ratione saltus amisso* ^DTR 25^
But if you start [to use] this formula at the month of September, after the manner of the Egyptians, whose year begins at that point, it is necessary that the Moon of July in that year have twenty-nine days and never more, one day having been removed because of the “leap of the Moon”. ( Wallis, 2004, 67)

DTR 25:

**Table:** 

**BVi. 4b44.58**	*egiptacdae .i. iiii kalendae*
	‘Egyptian i.e. the fourth calends’
**Ang. 58a37h**	*egiptii in .iiii. kalendis augustarum*
	‘of Egyptian in the fourth calends of August’
**BCr. 32b44**	*.i. hi.iiii. kalendis septembris*
	‘i.e. in the fourth calends of September’

I have already discussed these three glosses in-depth in Bauer (2017, 40–41). However, one thing I have overlooked there is the fact that both Ang. and BCr. have the preposition ‘in’ (in Latin and Old Irish respectively) preceding the Roman numeral ‘four’. Although the gloss is very hard to decipher in BVi., it seems that it has the Tironian note *.i.* at this position, because at least the first full stop of it can be read.

### XXI. Quae Sit Feria In Kalendas

CCSL 123B, 350

*… adde concurrentes sex, fiunt undecim: tolle* ^DTR 26^ *septem, remanent quattuor*
[…] add the 6 concurrents, and they make 11. Take away 7, and 4 remain. ( Wallis, 2004, 69)

DTR 26:

**Table:** 

**BVi. 4c43.70**	*cuire huait*
	‘Put from you!’
**Ang. 58b27**	*i. ot. a te.*
	‘Put from you!’
**BCr. 32c50**	*.i. cuire huait*
	‘Put from you!’

This is the second example Lambert (1983, 127) mentions as word-by-word correspondence between the Old Irish glosses found in BVi. and BCr. and the glosses in Ang. In contrast to DTR 2, however, Ang. has a bilingual gloss in this case. The conjugated Old Irish preposition *huait* – the second person singular of OIr. *ó* ‘from, out of, by’ – is transposed into the Latin phrase *a te* ‘from you’. It is worth mentioning that the same semantics are also expressed in a monolingual vernacular gloss a bit later in the Angers manuscript: *ot ti* ‘put from you’ (Ang. 59a12b). Furthermore, a gloss *âte* (Ang. 59a13e) is added to Lat. *tolle* ‘remove!’ only one line below. Hence, in the context of calculation instructions such phrases occur frequently. Accordingly, I feel hesitant to agree with Lambert without any doubt.

### XXII. Argumentum De Qualibet Luna Vel Feria

CCSL 123B, 352

*adde xiiii fiunt cxxviiii; partire per lviiii (quinquagies nouies bini cendecusoctus* ^DTR 27^ *), tolle cxviii, remanent xxviii.*
add 9, and that makes 129. Divide by 59: 59 times 2 is 118. Subtract 118 and 28 remain. ( Wallis, 2004, 70)

DTR 27:

**Table:** 

**BVi. 4d27.75**	*.i. a ocht deac ar chét*
	‘i.e. a hundred and eighteen’
**Ang. 59a13c**	*is eith nec guar cant.*
	‘it is hundred and eighteen’
**BCr. 32d40a**	*.i. a ocht deac ar chét*
	‘i.e. a hundred and eighteen’

In contrast to the two Old Irish glosses which have the numeral particle *a*, the gloss in Ang. features the third person singular present indicative of the copula in this position. Otherwise the glosses are very similar. This example is furthermore interesting because the parallel glosses do not appear in the exact same position within the base text. In BVi. and Ang. the glosses appear over Lat. *cendecusoctus* ‘one hundred and eighteen’ hence providing a vernacular translation of the somewhat hard to read long Latin number. In contrast to this, the gloss in BCr. appears in the intercolumnar space with a *signe de renvois* linking it to the same number which is depicted with Roman numerals just two words afterwards (cf. also Jones’ edition). It is also worth mentioning that *cendecusoctus* appears as *cendecus octus* at the line break in this manuscript. The gap caused by the continuation of the number in the next line makes the following interpretation possible. The glossator of the Karlsruhe Bede could have found the gloss in their exemplar and decided to put it into the intercolumnar space, because of the line break in the lemma it is glossing. The *signe de renvois* was only added later and since the spelled-out number is divided by the line break the glossator (or somebody else) did not look at the original lemma anymore, but decided that the gloss is for the Roman numeral *cxuiii* which appears as the third “word” in the line.

## Synthesis and results

**Figure 1.**
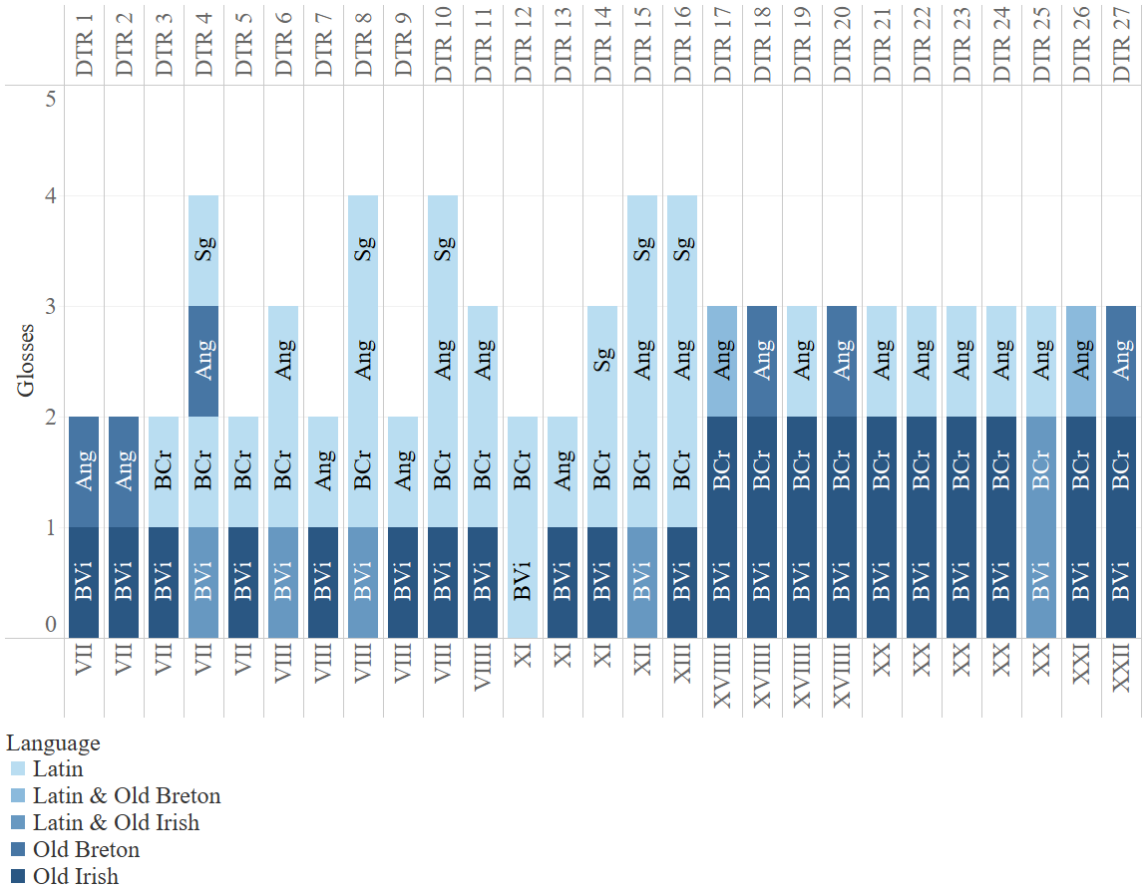
Distribution of the parallel glosses.

Figure 1 shows the distribution of parallel glosses between the different manuscripts and records their languages. What immediately strikes the eye is that there are no Old Irish parallel glosses before chapter XVIIII. This is caused by the fact that there are only three Old Irish glosses on DTR in BCr. before chapter XVII. ^19^ Parallel Old Breton glosses in Ang., on the other hand, exist throughout the chapters transmitted in the Vienna Bede (with a gap from chapter VIII to XIII – which does not mean that there are no Old Breton glosses within this range). It is furthermore interesting to see that the number of bilingual glosses is rather small: eight bilingual glosses stand against seventy monolingual glosses.

But what does the presented corpus tell us about whether the vernacular glosses are originals or translations? A first step is actually to look at the bilingual glosses. Table 1 gives an overview of them.

**Table 1.** The bilingual glosses in the corpus.

Example	Glossnumber(s)	Languages
DTR 4	BVi. 1a18.5	OIr. & Lat.
DTR 6	BVi. 1b27.10	OIr. & Lat.
DTR 8	BVi. 1c38.16	OIr. & Lat.
DTR 15	BVi. 3a29.32	OIr. & Lat.
DTR 17	Ang. 57b16e	OBret. & Lat.
DTR 25	BVi. 4b44.58 & BCr. 32b44	OIr. & Lat.
DTR 26	Ang. 58b27	OBret. & Lat.

We can see that most of the bilingual glosses (i.e. five) in our corpus appear in the Vienna Bede – Angers 477 has two instances and the Karlsruhe Bede only one. Taking a closer look at the data we see that in DTR 8, DTR 15, and DTR 25, the language switch happens before or after the Tironian note *.i.* which either stands for Lat. *id est* or OIr. *ed ón*. Bisagni (2013–2014, 26) rightly states that such instances should not be counted as code-switches, because “there is no way of establishing with certainty whether the Irish and the Latin section were composed at the same time, and by the same person.” This means that either the Irish or the Latin part might have been added at a later stage. Nonetheless, they are valuable cases for the present study, because at some point a glossator decided to add an Irish word or phrase to the otherwise Latin annotation.

For instance, it is interesting to note that while the Irish phrase in DTR 8 appears after the Latin part, DTR 15 and DTR 25 have the inverted order. And if we take a closer look at the latter two and compare them with the parallel glosses in Ang., we see that the latter manuscript has Latin words in the exact same position: Lat. *festis* vs. OIr. *feli* and Lat. *egiptii* vs. OIr. *egiptacdae*. Therefore, translation suggests itself. A direction for this is hard to determine – especially for DTR 15. In the case of DTR 25 we might tentatively suggest that Lat. *egiptii* in Ang. 58a37h goes back to OIr. *egiptacdae* in BVi. 4b44.58, because it does not appear in BCr. 32b44. This suggestion finds support in DTR 4. Similar to DTR 25, BVi. and Ang. also share parallels which are not present in the other two manuscripts here. Recalling Lambert's (1983, 128–129) statement from the introduction, in which he mentions a strong Irish influence on the glosses of Angers (especially the vernacular ones), we can interpret Ang. 50a7c *amestidiou* ‘circular courses’ as being a translation from OIr. *fithissi* ‘circular courses’. Which suggests that BVi. 1a18.5 is an original composition. In analogy to this, it could also be the case that (the beginning of) Ang. 58a37h in example DTR 25 is a translation from BVi. 4b44.58. Another peculiarity of the parallel glosses in this example which I have not mentioned in Bauer (2017, 40–41) is the fact that both Ang. and BCr. have the preposition ‘in’ preceding the Roman numeral *iiii* ‘fourth’ where BVi. has the Tironian note for ‘id est/ed ón’. As already mentioned above this is, however, not entirely clear to read. Hence it could be that the gloss in BVi. is a copy from another exemplar and the glossator misinterpreted OIr. *(h)i* ‘in’ as the Tironian note *.i.* (cf. BCr. 32b44). Similar to the previous examples, the BVi. gloss in DTR 6 also starts with Old Irish and then continues in Latin. A direction of translation, however, is not possible to determine in this case. The same is true for the two bilingual glosses found in Angers 477.

Turning to the monolingual glosses now, I will concentrate on those glosses in which BVi. has an Old Irish gloss and at least one of the other manuscripts has a Latin one. The examples in DTR 9 and DTR 16 show a closer connection between BVi. and Ang., in contrast to the other two manuscripts. This reflects the general observation that the glosses of BVi. and Ang. have more in common until chapter XIII. However, once the Old Irish glosses appear in the Karlsruhe Bede, i.e. after chapter XVII, BVi. and BCr. are more closely connected. Figure 1 visualises this very well, because we see that from chapter XVIIII onwards the two manuscripts always share parallel glosses in the same language and if one looks at the corpus presented above it shows that these are mostly verbatim. Further research is necessary to find possible reasons for the absence of Old Irish glosses in BCr. before folio 31 recto.

As already mentioned in the discussion of DTR 5 above, I am showing in a forthcoming publication that the Old Irish gloss is a translation from the Latin one. Since DTR 3 is quite similar in the sense that there are also two glosses only consisting of a verbal form, it seems plausible that BVi. 1a16.4 is also a translation from the Latin gloss BCr. 27a56. However, the peculiarities of the glosses mentioned above make any definite decision impossible. As already argued, a translation from Latin seems plausible for BVi. 2b28.25 in DTR 14. Influence the other way around, i.e. from Irish to Latin, and maybe even translation of the glosses can be seen in DTR 21. Irish influence on Latin or a translation from Irish might also lie behind the similar syntax in DTR 23. Scholars like O’Sullivan (2004, 84), Moran (2015, 141) and me ( Bauer, 2019b, 52) have stressed the need for extensive editions of glossed manuscripts and the glosses in DTR 12 and 19 show how important they actually are. Only with the help of the parallel gloss in BCr. it was possible to find the correct reading of BVi. 2a28.19a in the first example. In the second example the parallel gloss in Ang. helped to refine the analysis BVi. 4a2a.43 and BCr. 32a12. The form of the substantive verb in these glosses (OIr. *·mbí*) has been wrongly interpreted as third person habitual present in previous scholarship.

## Conclusions

There is no straightforward answer to the question of whether the vernacular Irish glosses are original compositions or translations from Latin glosses. They are in fact a bit of both. And this is not surprising, because it always needs to be kept in mind that the early medieval gloss corpora are not homogeneous (cf. Bauer, 2019d, 17), but were layered and copied over a long time-span. The different linguistic and thereby also chronological strata of the Old Irish glosses on Priscian’s *Ars Grammaticae* found in St Gall MS 904, for instance, have been the topic of several studies for more than a century now (cf. Lambert, 1996; Roost, 2013; Strachan, 1903) and yet no definite conclusions have been drawn to date. The issue of the genesis of the vernacular Celtic glosses is similarly complex. For some glosses, a translation from Latin into Irish is almost inevitable (cf. DTR 5 or DTR 14). Others, for instance DTR 21, suggest Irish influence on the Latin parallel glosses. A strong Irish influence on the Old Breton/Welsh glosses found in Ang. has already been stated by Lambert (1983) and cases like DTR 23 could show that this also extended onto the Latin glosses in this manuscript. Most of the cases, however, “only” show how closely related the Celtic manuscripts in the early medieval glossing tradition on the Venerable Bede’s *De Temporum Ratione* are. Since the Vienna Bede only survived the centuries as a fragment, the present article can only be seen as a pilot-study. Building on the presented methodology, more research should for instance be carried out on the Old Irish glosses in the Karlsruhe Bede and their potential Latin and Old Breton/Welsh parallels in Angers 477. This will bring further elucidation to the matters discussed here. Such studies, however, are only possible with extensive editions of early medieval manuscripts, including both vernacular and the Latin glosses. Only with such editions at hand will we be able to research the glosses as what they really are, a vital window into early medieval cultural and linguistic contact and the intellectual formation of Europe.

## Data Availability

Zenodo: Vernacular Parallel Glosses in the Gloss-ViBe corpus. https://doi.org/10.5281/zenodo.7936072 ( Bauer, 2023). This project contains the following underlying data: GlossViBe_Vernacular_Parallel_Glosses.csv Data are available under the terms of the Creative Commons Attribution 4.0 International (CC BY 4.0).

## Footnotes

^1^ Part of this study was presented at the Tionól 2022 (1819 November, 2022; Dublin Institute for Advanced Studies). I thank the participants for helpful input in the Q&A session and furthermore Jacopo Bisagni, David Stifter and Sean Winslow for invaluable advice and suggestions. All disclaimers apply.

^2^ “Pour nous, une bonne partie du commentaire d’Angers (notamment dans les gloses B) est empruntée à une tradition irlandaise” ( Lambert, 1983, 128–29). In my translation: ‘In my opinion, a good part of Angers’ commentary (notably the glosses [from hand] B) is borrowed from an Irish tradition.’

^3^ They are (with Wallis’ translation): VII. De Nocte ‘Night’, VIII. De Hebdomada ‘The week’, VIIII. De Hebdomadibus Septuaginta Propheticis ‘The seventy prophetic weeks’, XI. De Mensibus ‘The months’, XII. De Mensibus Romanorum ‘The Roman months’, XIII. De Kalendis, Nonis, Et Idibus ‘Kalends, Nones and Ides’, XIIII. De Mensibus Graecorum ‘The Greek months’, XV. De Mensibus Anglorum ‘The English months’, XVIIII. Item De Eodem Si Quis Computare Non Didicit ‘More on the same subject: for those who do not know how to calculate’, XX. Quota Sit Luna In Kalendas Quasque ‘What the age of the Moon is on any given first day of the month’, XXI. Quae Sit Feria In Kalendas ‘What day of the week it is on the Kalends’, XXII. Argumentum De Qualibet Luna Vel Feria ‘A formula for any Moon or weekday’.

^4^ https://digital.onb.ac.at/RepViewer/viewer.faces?doc=DTL_8650790&order=1&view=SINGLE (accessed 25 April 2023).

^5^ https://gams.uni-graz.at/o:glossvibe.bvi (accessed 25 April 2023).

^6^ See Barbet-Massin (2017) for an in-depth discussion of this manuscript.

^7^ Online at https://bvmm.irht.cnrs.fr/iiif/1097/canvas/canvas-375477/view (accessed 25 April 2023).

^8^ https://digital.blb-karlsruhe.de/id/20736 (accessed 25 April 2023).

^9^ See Bauer (2019c, 32–33) for a detailed discussion of the manuscript.

^10^ http://www.e-codices.unifr.ch/en/csg/0251/bindingA/0/Sequence-435 (accessed 25 April 2023).

^11^ The hyphens are inserted according to Lambert (1983, 122).

^12^ The gloss continues the quote of Isidore’s Etymologiae, but the following part is not important for the present discussion.

^13^ The quote is cited according to http://www.monumenta.ch/latein/text.php?tabelle=Isidorus&rumpfid=Isidorus,%20Etymologiae,%20Liber%2015,%20%20%208&domain=ldbfmmkmi&lang=1&von=verlinkung&inframe=1&hide_apparatus=1 (accessed 25 April 2023).

^14^ Bauer, Bernhard. Forthc. ‘Where Parallels Meet: Early Medieval Celtic and Latin Glosses’. In *Proceedings of Medieval Multilingual Manuscripts Conference*, edited by Nike Stam.

^15^ See DTR 19 below for a similar construction.

^16^ Cf. Wallis (2004, 43 fn. 121).

^17^ For a discussion of the usage of prepositions with dates in the glosses also see Bauer ( 2019a, 45; 2022, 172) or Stifter (2019).

^18^ The “.i.” is written much thinner and might not be part of the gloss at all. It is therefore excluded from the analysis.

^19^ See also two forthcoming publications: Bisagni, Jacopo. Forthc. ‘Glossing Time: Multilingualism and the Celtic Vernaculars in Early Medieval Computistical Manuscripts’. In *Proceedings of Medieval Multilingual Manuscripts Conference*, edited by Nike Stam and Bauer, Bernhard. Forthc. ‘“Although We Do Not See (It)”: Analysing the Latin Glosses of the Vienna Bede’. In *Margins at the Centre. Practices of Annotation, Scholars and Audiences in Carolingian East Francia*, edited by Cinzia Grifoni.
