# Supplementary material for: Early medieval vernacular Celtic glosses: originals or translations?  A case study on the Vienna Bede
Source: Open Res Eur. 2024 Mar 6;3:108. Originally published 2023 Jul 5. [Version 2] doi: 10.12688/openreseurope.16006.2 (PMC10521090; doi:10.12688/openreseurope.16006.2)
Supplement: Peer review history

# Reviewer report: Reviewer response for version 1

**DOI:** 10.21956/openreseurope.17283.r33548

**Published:** 2023-08-09

**Authors:** Pádraic Moran

**Affiliations:** 1 Classics (School of Languages, Literatures and Cultures), University of Galway, Galway, County Galway, Ireland

The article contributes to recent scholarship that investigates glosses on Latin manuscripts as evidence for early medieval multilingualism and language interaction. The corpus selected for study is clearly defined and well-organised. It expands previous work focusing on translation between Latin and Old Irish in the glosses by considering a third language, Old Breton. It also builds on an earlier study by Lambert (2005) by including an important third manuscript for Bede. The data is clearly presented and the analysis will certainly be of interest to scholars of Old Irish and/or Old Breton.

 The article needs improvement in the following respects:

 1) The argument is somewhat inconclusive, both in the analysis of each group of glosses and in the overall conclusion.

 For each group of glosses, the article should clearly state whether or not any information about direction of translation can be inferred. (This is left unclear e.g. for groups DTR 15, 17, 21, 22, 23, 25, 27. For DTR 17, even if the material is analysed in detail elsewhere, we need to know here what the conclusion is.)

 The overall conclusion should be more clearly presented. It begins with a very uncertain tone (‘a bit of both’), but eventually turns to stronger evidence (‘a translation from Latin to Irish is almost inevitable’). It would be more useful to consider the strongest evidence first, then to consider its implications, before adding caveats. The final discussion of future possible research should be in a separate paragraph, for clarity.

 2) The following are issues regarding interpretation:

 DTR 1: Gloss is surely intended for *caeca*, not *nocte*?

 DTR 2: ‘although we do not see (it)’—why is ‘it’ in brackets, when there is clearly an infixed pronoun?

 DTR 3: *adrigiter *is presumably a typo for *ardrigiter*?

 If a relative, is the translation ‘by which they appear’ necessary? Why not just ‘which appear’? This could explained if the author fronted *sidera *in his (mental) translation: ‘stars which appear to be shining lights’.

 DTR 4: ‘in Greek, circle is translated as apside’ should be ‘in Greek, *absida *is translated as circle’ (for better clarify and mirroring the other translations following).

 ‘While Gr. αψίδα is usually only used to denote the architectural structure, the related form Gr. ἁψῑ́ς can also mean ‘wheel, hoop, disc’.’ This is very confusing. They are presumably the same word ἁψίς (ἁψίδα is acc. sg.), which easily encompasses all of these meanings. There is no need for a separation here. The confusion really arises in Isidore (who says he is not sure what the nominative should be).

 ‘A connection with circle is therefore not too far-fetched.’ Far from being far-fetched, it is the core meaning. The connection with ‘bright’ is the far-fetched one, since it is an invention by Isidore to service an etymology.

 CCSL 123B, 303–304: Meaning of *iubebantur *is missing from translation.

 DTR 8: Latin nouns in oblique cases are supplied with prepositions elsewhere (e.g. DTR 9, 15, 16) so *uno/una (die) *should be here too (‘with one day’?).

 ‘one Sunday’ for *una die dominico *seems inadequate. Suggest ‘with one day, a Sunday’ (which more closely parallels the other glosses).

 Does we have to assume a progression from simple to more complex? What about the other possibility, that glosses were abbreviating their source material? Surely that’s equally likely?

 DTR 9: ‘Since Old Irish does not have an ablative case…’. That is not relevant in this situation. The Latin lemma *collectis *‘after being collected’ is indeed ablative, but the Irish gloss is not a substitution gloss. It instead provides supplementary information (‘[collected] for an offering’), so does not need to mirror the case of the lemma.

 DTR 10: *mundari *‘cleansed’ > ‘to be cleansed’ × 2

 DTR 11: ‘endicad’ in translation is not an English word; should be ‘hendecad’. (‘Octad’ is OK, though ‘ogdoad’ seems more common in writing on computistics.)

 DTR 15: *festis *‘feasts’ > ‘on the feasts’ (since *feriae *below is ‘of the … feast’).

 DTR 20: *dind leth ailiu* (× 2) is idiomatic for ‘on the other side’; see *eDIL *s.v. *leth *IV (c) (and cf. Latin *ex parte*). In which case, the distinction of prepositions discussed below seems not significant: they are both saying the same thing.

 DTR 24: ‘in contrast to’—suggest reword, since BCr. is just slightly more specific, not really contrastive.

 3) Various minor errors and inconsistencies need to be corrected:

 p. 3:

 ‘Corpus Christianorum Series Latina, i.e. Jones, 1977’—insert ‘123B’ after ‘Latina’.

 note 1: ‘1819’ > ‘18–19’

 ‘the Anger 477s’—delete ‘the’

 p. 4:

 ‘chapter’ > ‘section’ × 2

 ‘lemma’ (as plural) > ‘lemmata’ or ‘lemmas’

 ‘where chosen, because’—delete comma

 ‘late 8th/early 9th century’ > ‘late eighth/early ninth century’ (as elsewhere)

 ‘either originates from Brittany or North-East France’ > ‘originates from either Brittany or North-East France’

 ‘cf. Bronner, 2013’—insert page reference.

 ‘Latin of Old Breton’—‘of’ > ‘or’

*VII. De nocte*—lemmata in Latin text should be underlined here, as elsewhere

*Lunam uero… fuscari*—a translation for this final part is missing.

 p. 5: ‘in the latter manuscript’—insert comma after (to mark off adversative clause)

 p. 7: ‘CCSL 132B’ > ‘CCSL 123B’

 p. 8: ‘CCSL 132B’ > ‘CCSL 123B’

 p. 12: ‘Glossnumber(s)’ > ‘Gloss number(s)’

 p. 14: Reference for Roost needs more information.

 Figure 1: The colours in the centre of the range are very hard to distinguish. Recommend to use more contrastive colours for clarity.

 Other: Better to omit all macrons and breves from Latin and Greek words, rather than use them inconsistently: p. 5 ἁψῑ́ς, p. 7 *lūstrārī, lūstrāre, mundāre*, p. 8 *lūnāris*, ἐπῐ́

If any, are all the source data and materials underlying the results available?

Yes

Is the work original in terms of material and argument?

Yes

Is the work clearly and cogently presented?

Partly

Is the argument persuasive and supported by evidence?

Partly

Does the research article contribute to the cultural, historical, social understanding of the field?

Yes

Does it sufficiently engage with relevant methodologies and secondary literature on the topic?

Yes

Reviewer Expertise:

Latin and Old Irish glosses

I confirm that I have read this submission and believe that I have an appropriate level of expertise to confirm that it is of an acceptable scientific standard, however I have significant reservations, as outlined above.

# Reviewer report: Reviewer response for version 1

**DOI:** 10.21956/openreseurope.17283.r33549

**Published:** 2023-08-01

**Authors:** Chantal Kobel

**Affiliations:** 1 School of Celtic Studies, Dublin Institute for Advanced Studies, Dublin, Ireland

An interesting article that seeks to address the difficult question of the genesis of the vernacular glosses on Bede’s *De Temporum Ratione*. Well worth publishing. This is a good case study of a small corpus of material which sets out the data clearly, followed by a detailed discussion of the results. The following comments are intended to help strengthen certain points in the article, followed by some recommended minor stylistic changes.

**Comments**:

 p. 4a: What is the relationship between the four copies of glosses? Perhaps a note following the description of the manuscripts would be helpful here. Cf. the comment on p. 13a ‘the gloss in BVi. is a copy from another exemplar …’. Does this imply that other shared glosses between BVi./ Ang. BCr. are from the same exemplar?

 p. 4b/5a: There is no English translation corresponding to the Latin text beg. ‘Lunam uero aiunt … memorata fuscari’ provided. Double check that all other Latin passages are provided with full translations from Wallis.

 p. 5a ‘BVi. 1a9b1 dorchai’. Should the complete gloss ( *dorchai nocte*) be given here to fully express the adjectival force in this instance and that it is not misconstrued as a substantivised adjective? This is what is printed in *Ériu* 67 (2017, 31) after all.

 p. 5a ‘BVi. 1a16.4 adrigiter’ > recte *ardrigiter*.

 p. 7b7.8: add reference to *Thesaurus Palaeohibernicus* here for the former reading of *i mbe*.

 p. 7b20 ‘but it is very likely the Tironian note .i.’. Yes, I would agree. In fact, I can make out the letter *i* and following punctum, with only the first punctum obscured by the crease in the manuscript.

 p. 8a1 ‘BVi. 2a35.22 .i. reim ṅgrein’. It would be worthwhile having a note here on the form *ṅgrein*, which is translated as a gen. sg.! Is this the correct reading? Thes. Pal. has *ngreine*.

 p. 9a16 ‘As I have **in** stated elsewhere’ delete **in**.’

 p. 10b46 ‘However, one thing I have overlooked there is the fact’ Suggestion > ‘However, I overlooked the fact’.

 p. 11a1 ‘it seems that it has the Tironian note .i. at this position, because at least the first full stop of it can be read’. Is that really the case? If only the first stop can be read (and without following *i.*), what’s not to say that this is a full stop? I find most of this gloss is almost entirely illegible apart from the final abbreviation for *kalendae*. This should be made clear here in the first instance, rather than on p. 13.

 p. 12b14 ‘Which suggests that BVi.’ > suggestion ‘This suggests that …’.

 p. 12b20 ‘where BVi. has the Tironian note for *id est*’. But this reading is unclear in the manuscript (which you do mention on the following page). Perhaps one should be more circumspect here. Also, could the exemplar have had *i* + *n*-stroke for Lat. *in*, like in Ang.? The n-stroke may have been lost in transmission.

 p. 13a ‘O’Sullivan (2004, 84), Moran (2015, 141) and me (Bauer 2019b, 52’)’ > ‘O’Sullivan (2004, 84), Moran (2015, 141) and Bauer (2019b, 52)’

**Some minor stylistic points**:

 Avoid the use of the passive, e.g. p. 4 ‘The two final chapters are synthesising the results’ > ‘the two final chapters synthesise the results’; p. 5a24 ‘I am showing’ > ‘I show’.

 Some sentences are quite long, extending over four lines of text, see for instance p. 11 col. b beg. ‘The *signe de renvois* was only added …’ (this sentence has over 50 words!). Rewrite as shorter sentences for ease of reading.

 Footnote numerals are placed inconsistently before and after punctuation. See for instance on p. 4a22, 25, 27 versus p. 4a34, 4b4 etc. Ensure all are placed following the full stop. Also, incorporate footnote 4 and 5?

If any, are all the source data and materials underlying the results available?

Yes

Is the work original in terms of material and argument?

Yes

Is the work clearly and cogently presented?

Yes

Is the argument persuasive and supported by evidence?

Yes

Does the research article contribute to the cultural, historical, social understanding of the field?

Yes

Does it sufficiently engage with relevant methodologies and secondary literature on the topic?

Yes

Reviewer Expertise:

Old Irish; Palaeography

I confirm that I have read this submission and believe that I have an appropriate level of expertise to confirm that it is of an acceptable scientific standard, however I have significant reservations, as outlined above.

# Reviewer report: Reviewer response for version 1

**DOI:** 10.21956/openreseurope.17283.r33551

**Published:** 2023-08-01

**Authors:** Aaron Griffith

**Affiliations:** 1 Utrecht University, Utrecht, Utrecht, The Netherlands

The article examines early vernacular Celtic glosses and tries to answer the question as to whether they are original compositions or translations. This is a large and complicated question, so the article examines it within a limited corpus, that of the Vienna Bede. The question is also part of a larger discourse on glossing and glossing contexts, and this is indicated appropriately via citations of other relevant works and in the general approach. 

 The Vienna Bede is only a fragment, so there is a limited amount of data available to analyse. The conclusions are therefore quite modest. Nonetheless, there are some valuable observations and emendations suggested for individual glosses in the corpus (e.g. DTR 12 and 19). This article brings together such corrections with a well-informed overview of the situation surrounding glossing on DTR in a Celtic context.

 The data presentation and analysis is brief, but generally clear, and the same goes for the synthesis. For the bilingual glosses under examination, there are a number of observations and suggestions for interpretation. These are all plausible, though not forcing (the author is clear on this, however, and is not over-interpreting the data). I do not think that stronger conclusions are possible or warranted at this time. The author's plea for further complete editions of texts is fully-justified and a very reasonable end to the article. The article is a relatively short contribution to existing scholarship, but it presents results commensurate with that and is worthy of publication.

 A number of specific comments follow (referring to page number and column letter):

 Page 3b bottom: “The present study goes one step further back in the Celtic glossing tradition on Bede’s *De Temporum Ratione*. It takes the Vienna Bede (= BVi.) as its main source, to research the genesis of the Old Irish glossing tradition on Bede’s computistical work which influenced the Angers 477’s (vernacular) glosses to a large extent.” There is a bit of ambiguity in this statement. Is the assumption that BVi is the primary source for Anger 477? Or is this to be proven?

 Page 4a top: for “chapter”, rather “section”?

 Page 4a: DTR3: Is BVi 1a16.4 mistyped? Should it not read *ardrigiter* ‘by which they appear’ (as in the main text)? Why is it translated as a prepositional relative instead of a regular relative ‘which appear’?

 Page 5a top: The explanation offered of Old Irish *con·destis* is presumably a concise summary of the argument in Bauer, forthcoming. Could this perhaps better be indicated directly?

 Page 9a: DTR 17: though there is a reference to a full discussion elsewhere, it would be good to give some indication to the reader what one should take away from this gloss: direct connection but unclear direction? likely but unclear connection?

 Page 12: Figure 1 presentation: The figure is effective in representing the data. I wonder if it would be more effective, however, if the manuscripts each got their own row in the data (with BVi on the bottom as now, then a row for BCr, then Ang, then Sg), rather than simply stacking them up as now. It would make it harder to see which individual DTR number had how many glosses (since there would now be gaps in the table), but since the numbers are low, this would not be a significant hindrance. The added benefit would be that it would be easier to see what individual mss. were doing with respect to the language of the parallel glosses under discussion.

 Page 13a: When turning to the more numerous monolingual glosses, I wonder where some of the generalisations are coming from. That is,  it is stated: “This reflects the general observation that the glosses of BVi. and Ang. have more in common until chapter XIII. However, once the Old Irish glosses appear in the Karlsruhe Bede, i.e. after chapter XVII, BVi. and BCr. are more closely connected.” It is not clear to me where these observations are derived from. The data visualisation indeed is in agreement with these obsevations, but is this also the evidence or is it intended as confirmation of others’ work?

 Page 13b: To the references in the layers in the Sg. glosses, one might now add Griffith 2022 and Meng 2023. The statement in the text remains true, however.

If any, are all the source data and materials underlying the results available?

Yes

Is the work original in terms of material and argument?

Yes

Is the work clearly and cogently presented?

Yes

Is the argument persuasive and supported by evidence?

Yes

Does the research article contribute to the cultural, historical, social understanding of the field?

Yes

Does it sufficiently engage with relevant methodologies and secondary literature on the topic?

Yes

Reviewer Expertise:

Early Irish, Middle Welsh, historical linguistics, language change

I confirm that I have read this submission and believe that I have an appropriate level of expertise to confirm that it is of an acceptable scientific standard.

## References

[1] : Examining linguistic layers in the St. Gall glosses, again. *North American journal of Celtic studies* .2022;6(1) : 10.1353/cel.2022.0004 143-160 10.1353/cel.2022.0004

[2] : A Clustering Approach to Uncover Hidden Layers of Glosses in the St. Gall Priscian. *Utrecht University BA thesis.* .2023;
